# ChatGPT as a self-regulated learning support tool in thesis writing: a mixed-methods study

**DOI:** 10.3389/fpsyg.2026.1871579

**Published:** 2026-09-17

**Authors:** Nagaletchimee Annamalai, Chuhan Niu, Xiaofan Wu, Amer Ayada Ayoub Alkubaisy

**Affiliations:** 1 Department of Languages and Culture, College of Humanities and Sciences, Ajman University, Ajman, United Arab Emirates; 2 Humanities and Social Sciences Research Center (HSRC), Ajman University, Ajman, United Arab Emirates; 3 School of Languages, Literacies & Translation, Universiti Sains Malaysia, Minden Heights, Malaysia; 4 School of Distance Education, Universiti Sains Malaysia, Minden Heights, Malaysia

**Keywords:** artificial intelligence, ChatGPT, higher education institutions, self-regulated learning, writing skills

## Abstract

This study examines the potential of ChatGPT as a writing tool for postgraduate students, emphasizing its role in planning, monitoring, and evaluation. Guided by Zimmerman's Self-Regulated Learning (SRL) framework, this study investigates how ChatGPT supports postgraduate students during the forethought, performance, and self-reflection phases of thesis writing. Using a mixed-method research design, data from 303 postgraduate students through a survey and 20 interviews highlight various positive practices and concerns among postgraduates. The quantitative findings revealed that students reported positive experiences using ChatGPT to support planning, monitoring, and evaluation during thesis writing. The qualitative findings further demonstrated that ChatGPT assisted students in outlining chapters, brainstorming ideas, clarifying research objectives, verifying information, refining prompts, and critically evaluating AI-generated content. The sub-themes indicate that evaluation within the SRL cycle was characterized by strong metacognitive control, critical judgment, and authorial agency. Participants consistently positioned themselves as primary authors, using AI as a reflective and supplementary tool rather than a replacement, thereby safeguarding academic identity and scholarly contribution. Overall, the findings suggest that ChatGPT can function as a cognitive and metacognitive scaffold that supports self-regulated thesis writing when used critically and strategically. The study provides critical implications for leveraging ChatGPT among postgraduates to enhance their writing skills while maintaining academic integrity and authorial ownership.

## Introduction

In recent years, the development of artificial intelligence (AI) has significantly transformed instructional practices at higher education institutions worldwide (Chiu, 2024b). International organizations such as UNESCO have highlighted Artificial Intelligence as the theme for International Day of Education 2025 (UNESCO, 2025). AI can “fundamentally alter the way we instruct and acquire knowledge” (Aziz et al., 2024).

Empirical studies have demonstrated that students are optimistic about employing AI in their learning environment to enhance their academic skills and to establish effective learning approaches (Annamalai, 2025; Annamalai and Nasor, 2025; Hsu and Hsu, 2025). Proponents argue that AI tools are practical to integrate into teaching and learning activities, given their abilities to generate ideas, draft, edit, and detect grammatical, sentence structure, and syntactic problems (Annamalai and Nasor, 2025; Kohnke et al., 2023). They are also appreciated for their effectiveness in providing basic knowledge on various subjects (Tlili et al., 2023). As AI becomes increasingly important in students' lives, it becomes pertinent to understand how students employ AI tools, particularly in research writing.

Although existing studies provide meaningful insights, they remain limited in providing a comprehensive understanding of AI tools. Beyond examining the effectiveness of AI in supporting academic tasks, there is a growing need to understand how AI influences learners' cognitive and metacognitive processes during complex academic activities such as thesis writing. According to Tekir (2026), most existing studies focus on linguistic aspects related to fluency, accuracy, and cohesion, which normally generate positive outcomes. However, considerably less attention has been devoted to understanding how students use AI to plan, monitor, and evaluate their writing processes, which are central components of self-regulated learning. Limited studies demonstrate the deeper dimensions of writing and monitor their relationship to idea generation, critical arguments, and learners' ability to regulate and monitor their writing. Debates related to cognitive offloading, authorship, and learners' experiences and reflections on their use are particularly limited in the higher education context (Tekir, 2026). It is pertinent for learners to acquire the appropriate knowledge related to the limitations of AI and how to use AI wisely to gain maximum benefits in their learning activities (Xia et al., 2024). A major concern is also the susceptibility of AI tools to hallucination, which undermines their trustworthiness and reliability in an educational environment (Ji et al., 2023; Annamalai, 2025).

Such framing restricts deeper exploration of how student–AI engagement may fundamentally reshape or influence learners' behavioral patterns, cognitive development, and regulatory capacities (Popenici and Kerr, 2017; Păvăloaia and Necula, 2023). Consequently, Self-Regulated Learning (SRL) provides a useful theoretical lens for examining how learners manage their cognitive, motivational, and metacognitive processes while interacting with AI tools. According to Zimmerman (2000), SRL refers to learners' ability to proactively plan, monitor, and evaluate their learning in pursuit of academic goals. SRL enables individuals to keep pace with technological advancements and serves as a vital component in the effective and responsible use of AI (Markauskaite et al., 2023). According to Jivet et al. (2020), to effectively assess the use of AI tools that support SRL, it is pertinent to examine learners' experiences with such applications. Despite the growing adoption of ChatGPT in higher education, limited empirical research has examined how postgraduate students employ ChatGPT across the forethought, performance, and self-reflection phases of SRL during dissertation writing. Guided by SRL, this study employs a mixed-methods explanatory design to examine postgraduate students' use of ChatGPT in completing their dissertations.

Dissertation writing is one of the most challenging skills and represents one of the most demanding academic undertakings, requiring sustained cognitive effort, effective strategies, emotional regulation, and self-regulatory competence (Kim et al., 2025). Many postgraduates face challenges related to motivation, procrastination, ineffective time management, psychological problems, limited knowledge of research writing, and limited supervisory support (Nurkamto et al., 2023). Studies examining postgraduate thesis writing strategies remain scarce, particularly in recent years regarding GenAI (Kessler, 2020). The current study seeks to address this gap by investigating how postgraduates employ SRL in completing their dissertation for various chapters. Understanding postgraduate practice is significant for developing effective pedagogical interventions, supervision practices, and institutional support.

This study contributes to the literature in three ways. First, it provides empirical evidence regarding how postgraduate students utilize ChatGPT throughout the dissertation writing process. Second, it extends current understanding of SRL in AI-mediated learning environments by examining how ChatGPT supports planning, monitoring, and self-reflection activities. Third, the findings offer practical recommendations for supervisors, instructors, and higher education institutions seeking to promote responsible and effective AI integration in postgraduate research writing. The findings are also significant for the development of the SRL framework that considers interactions between AI and humans. Recommendations to higher education institutions are also possible, including suggestions for implementing appropriate interventions and workshops to enhance students' capacity and enable them to complete their dissertations effectively with appropriate competence and long-term scholarly development.

Recent studies have reported that ChatGPT facilitates the SRL process (Wu et al., 2023; Xia et al., 2024). During the planning phase, students need to be motivated and engaged in their learning activities (Chiu, 2024a). During the monitoring phase, it can be used to support learners' motivation in their learning (Wu et al., 2023). During the evaluation phase, students can request feedback from ChatGPT for suggestions and to clear their doubts in their learning process (Kasneci et al., 2023). While these studies suggest the potential of ChatGPT to support SRL, most focus on general learning activities rather than the complex and extended process of dissertation writing. Furthermore, few studies have explored how postgraduate students experience and utilize ChatGPT across all three phases of Zimmerman's SRL framework. However, according to Chiu (2024a), studies related to SRL and ChatGPT are limited. Therefore, this study addresses an important gap by examining how ChatGPT assists postgraduate students during the forethought, performance, and self-reflection phases of dissertation writing.

The research questions for this study are:

**RQ1:** What is the level of students' self-regulated learning (forethought, performance, and self-reflection) when using ChatGPT during thesis writing?
**RQ2:** How does ChatGPT assist students in the forethought, performance, and self-reflection phases while preparing for thesis writing?

## Theoretical framework

SRL has been widely accepted as an important factor influencing learners' academic success. Based on social cognitive theory, SRL refers to an individual's active planning, monitoring, and evaluating their cognitive, motivational, and behavioral processes to achieve specific learning goals. According to Zimmerman (2000; 2002), self-regulated learning is a process in which an individual utilizes one's mental abilities to develop academic skills through self-directed learning. SRL defines learning as a cyclical, purposive set of self-directed approaches that focuses on planning (*forethought)*, monitoring (*performance), and evaluation* (*self-reflection)*. Learners' ability to effectively manage their self-regulated learning (SRL) is pertinent for their continuous growth and independent learning in today's digital world (Chen and Huang, 2014). To encourage learners' SRL behaviors, studies have also suggested decomposing learning strategies by providing opportunities for learner autonomy, encouraging goal setting, and providing feedback that supports and eventually creates a positive learning environment (Zhang et al., 2025).

Zimmerman's (2000; 2002) SRL framework provides a useful lens for understanding how ChatGPT may support postgraduate students during dissertation writing. In the forethought phase, learners engage in goal setting, strategic planning, task analysis, and self-motivation. ChatGPT can support this phase by helping students generate research ideas, refine research questions, organize writing plans, and clarify complex concepts. During the performance phase, learners monitor their progress, apply learning strategies, and regulate their efforts while completing tasks. ChatGPT can facilitate this process by providing real-time explanations, answering questions, suggesting revisions, and offering immediate feedback that enables students to monitor and adjust their writing strategies. In the self-reflection phase, learners evaluate their performance, assess the effectiveness of their strategies, and identify areas for improvement. ChatGPT can contribute to this phase by providing feedback on drafts, highlighting weaknesses in arguments, and prompting students to critically reflect on their writing decisions.

Accordingly, this study conceptualizes ChatGPT not merely as a writing assistant but as a metacognitive support tool that may facilitate the three cyclical phases of SRL. The framework assumes that interactions with ChatGPT can enhance students' ability to plan, monitor, and evaluate their dissertation-writing processes, thereby supporting more effective self-regulated learning. This conceptual relationship forms the foundation of the study and guides the development of the research questions, survey instrument, data analysis, and interpretation of findings. In this study, the theoretical framework guides the construction of survey questions, analysis and interpretation of the results by examining how ChatGPT supports postgraduate students' planning, monitoring, and evaluation activities during dissertation writing.

## Studies related to SRL

Previous studies have documented that students who strategically employed SRL strategies exhibit stronger academic performance, greater autonomy, and positive learning outcomes. Bai (2015) has indicated that well-designed SRL interventions can enhance learners' writing. Also, SRL has been reported to improve writing by fostering learners' language-related self-efficacy (Teng and Zhang, 2020). In recent studies, Shen and Bai (2024) developed a framework that guides learners to regulate their engagement with AI to develop their writing processes. Although prior studies have examined SRL and AI-assisted learning separately, increasing attention has been given to understanding how generative AI tools such as ChatGPT can support specific SRL processes. Drawing on Zimmerman's SRL framework, ChatGPT can be conceptualized as a metacognitive scaffold that assists learners across the forethought, performance, and self-reflection phases. Through its conversational and feedback-oriented features, ChatGPT has the potential to facilitate goal setting, strategic planning, progress monitoring, and reflective evaluation, all of which are essential components of self-regulated learning.

A number of researchers have highlighted that ChatGPT can strengthen SRL. Baidoo-Anu and Ansah (2023) conducted an exploratory study and found that ChatGPT promotes personalized and interactive learning. The study also emphasized ChatGPT's limitations, including the generation of inaccurate and biased information. Chiu (2024a) asserts that ChatGPT enhances three important components of SRL: planning, monitoring, and evaluating. Wu et al. (2023) reported that ChatGPT successfully answered students' questions and doubts, which eventually developed their SRL. From the perspective of the forethought phase, ChatGPT can support learners in establishing goals, generating ideas, organizing tasks, and developing writing strategies. For example, students may use ChatGPT to brainstorm dissertation topics, refine research questions, or create writing schedules. Such activities align with the planning and goal-setting processes described in Zimmerman's SRL model.

In recent years, Ng et al. (2024) examined how ChatGPT enhanced science education. The experimental study employed SRLbot and rule-based AI chatbots (Nemobot). The study found that SRL guided students to acquire science knowledge effectively. The ChatGPT instructions minimize anxiety and encourage learning. Lee et al. (2024) employed a guidance-based ChatGPT-assisted Learning Aid, encouraging students to solve learning problems independently before seeking assistance from ChatGPT. The findings were positive, and students were developing SRL and higher-order thinking skills. These findings suggest that ChatGPT may play a significant role during the performance phase of SRL by supporting ongoing monitoring and strategy implementation. The immediate feedback and explanations provided by ChatGPT allow learners to identify knowledge gaps, clarify misunderstandings, and regulate their learning strategies while completing academic tasks. Such interactions can strengthen metacognitive awareness and facilitate continuous monitoring of learning progress.

Zhu (2025)) found that ChatGPT contributes to self-regulation across planning, monitoring, and evaluation by assisting learners in planning their goals, managing time, enhancing motivation and helps in time management. Also, the evaluation phase provides feedback so that learners are able to evaluate and make changes to their learning process. Similarly, during the self-reflection phase, ChatGPT can provide formative feedback on written work, suggest improvements, and encourage learners to assess the effectiveness of their writing strategies. By comparing their drafts with AI-generated suggestions, students may engage in reflective judgement and identify areas requiring further revision. Therefore, ChatGPT can be viewed as a tool that supports reflective evaluation and continuous improvement within the SRL cycle.

A number of studies have also examined postgraduate students. For example, Zhang et al. (2026) examined PhD candidates using Activity Theory. The findings reported on the hybrid approach of writing a dissertation. Participants in this study employed AI for translation, summarization and stylistic refinement. Another study by Krumsvik (2025) found that acceptance of AI among PhD supervisors is limited, and they expressed dissatisfaction with ethical considerations. Zerey and Özmen (2026) conducted a study with MA students on thesis writing and identified challenges related to research competence, psychological stress and bureaucracy that hinder the dissertation completion. While these studies demonstrate the growing use of AI in postgraduate research and writing, they provide limited insight into how AI supports specific SRL processes throughout dissertation writing. However, these studies do not consider SRL.

Taken together, the reviewed studies suggest that ChatGPT possesses features that align with all three phases of Zimmerman's SRL framework. Nevertheless, empirical evidence explaining how postgraduate students utilize ChatGPT to support planning, monitoring, and evaluation during dissertation writing remains limited. Existing studies predominantly focus on learning outcomes or general AI adoption rather than examining the underlying self-regulatory mechanisms through which ChatGPT may influence academic writing processes.

The above studies have witnessed a growing scholarly effort to integrate SRL into the educational context, with no sign of slowing down. SRL is viewed as significantly influencing cognitive, metacognitive, motivational, and behavioral strategies to achieve effective learning objectives. Collectively, the literature indicates that ChatGPT may function as a metacognitive support tool that facilitates planning through idea generation and goal setting, monitoring through immediate feedback and guidance, and evaluation through reflective feedback and revision support. However, this conceptual relationship has not been sufficiently examined among postgraduate students engaged in dissertation writing.

Despite the positive findings, several limitations remain evident in the existing literature. Firstly, most of these studies focused on undergraduates and used short-term interventions without an in-depth investigation of SRL in a controlled classroom setting. Postgraduate students tend to demonstrate a high level of critical thinking and independent learning, particularly in dissertation writing, as an extended academic writing. The studies related to postgraduate studies, particularly in writing their dissertations, are limited. Secondly, existing studies emphasize AI-supported learning outcomes, such as improvements in text quality, coherence, and grammar, rather than investigating the writing and regulatory mechanisms. In dissertation writing, it is vital to understand sustained cognitive engagement and SRL. Furthermore, the use of AI is accompanied by several limitations. For example, concerns include the generation of inaccurate information (Annamalai and Nasor, 2025), academic dishonesty (Tlili et al., 2023), and difficulties in determining the accuracy of AI-generated content (Tlili et al., 2023).

Furthermore, it is also pertinent to carefully consider potential challenges that learners may encounter when implementing AI applications in actual settings, including privacy infringements, algorithmic biases, and surveillance (Seo et al., 2021). These limitations underscore the need for further research on how AI can effectively support deep learning.

## Methodology design

This study applied a sequential explanatory mixed-methods design, in which a quantitative study is followed by a qualitative phase (Creswell et al., 2003). The quantitative phase involved a survey that provided a general sense of how postgraduate students use ChatGPT in their dissertations. The qualitative phase was conducted after the quantitative phase to clarify and understand the quantitative data. Twenty survey respondents who volunteered to participate in follow-up interviews were recruited to provide detailed explanations of how they used ChatGPT during thesis writing. Quantitative and qualitative findings were merged through a joint display that compared statistical patterns with qualitative themes and illustrative evidence. Then, inferences were developed in the discussion by identifying areas of convergence, complementarity, and divergence between the two data. By leveraging quantitative and qualitative data, the study gained an in-depth understanding of ChatGPT in completing their dissertations.

### Participants and selection

The study included 303 postgraduates from a higher education institution in northern region of Malaysia. The participants' ages ranged from 25 to 50 years. There were 159 female participants and 144 male participants. The countries of origin of these students were China and Malaysia (China *n* = 200; Malaysia *n* = 103). These universities offer social science and science courses for postgraduate students. Purposive sampling was employed. Students were briefed on the nature of the study, the questionnaire and the interview questions. They were also informed that they could exit from this study at any time without any penalty. All participants were given pseudonyms (T1, T2, T3….) and data collected will only be used for research purposes and will not be revealed to a third party.

Postgraduates were chosen for the current study because writing academic material in English is a challenging task for many non-native speakers from different countries. These participants are from China and Malaysia, and English is a second language for them. Table 1 presents the demographic information of the participants.

**Table 1:** Demographic profile of respondents.

Variable	Category	*n*	Percentage
Gender	Female	159	52.5**%**
	Male	144	47.5**%**
Country	China	200	66.0**%**
	Malaysia	103	34.0**%**
Age group	≤ 25	28	9.2**%**
	26–30	91	30.0**%**
	31–35	124	40.9**%**
	36–40	32	10.6**%**
	>40	28	9.2**%**
Year of study	Year 1	28	9.2**%**
	Year 2	99	32.7**%**
	Year 3	124	40.9**%**
	Year 4 or above	52	17.2**%**

### Instrument and data analysis

#### Questionnaire

A questionnaire was developed using Google Form and distributed to the participants in the university where the investigation was conducted. The questionnaire was designed to examine postgraduate students' use of ChatGPT to guide them in their dissertations. In the questionnaire, the nature of the study was explained, potential benefits and risks, the rights of the research participants and the context of the researchers for this survey. Out of the 303 surveys, 20 participants were invited. The participants who volunteered.

The questionnaire consists of two sections, totalling 21 items. The first section gathered information on gender, age, country of origin, years of study, and institutions. The second section includes items related to SRL. Participants rated each item on a 5-point Likert scale (1 = strongly disagree to 5 = strongly agree). The statistical analysis was carried out by employing IBM SPSS Statistics Version 27 (IBM Corp., Armonk, NY, United States). The survey items were developed based on the literature review conducted for the study. The survey items related to self-regulated learning were adapted from Zhang and Wang (2025), Barnard et al. (2009), and Noels et al. (2000). The adaptations focused on aspects of planning, monitoring, and evaluation. Item development followed a construct-driven procedure anchored in Zimmerman's (2000) cyclical SRL model. Each of the three theoretical phases was operationalised as a distinct sub-scale: planning items captured goal setting, outlining, and task structuring (items 1–5); monitoring items captured progress checking, comparison, and prompt adjustment (items 6–10); and evaluation items captured critical appraisal, verification, and authorial ownership (items 11–15). Source items were adapted to the ChatGPT thesis-writing context by re-anchoring each stem to a concrete AI-assisted behavior, and the mapping between every item and its underlying construct was reviewed by the two subject-matter experts to establish content validity before piloting.

Internal-consistency reliability was estimated using Cronbach's alpha for each subscale. In the present sample (*N* = 303), alpha was 0.69 for planning,0.67 for monitoring, and 0.70 for evaluating, and 0.86 for the 15-item composite, indicating acceptable reliability at the scale level. Because coefficient alpha establishes internal consistency but not dimensionality, construct validity was further examined through confirmatory factor analysis (CFA) of the hypothesized three-factor structure (planning, monitoring, evaluation), with composite reliability (CR) and average variance extracted (AVE) computed for each latent factor. Two experts in education and technology reviewed the questionnaire for face validity, and their comments were used to revise the items.

#### Quantitative analysis plan

Quantitative data were analyzed in IBM SPSS 27 and cross-validated in the semopy and statsmodels libraries. Analyses proceeded in four stages aligned with RQ1. First, descriptive statistics were computed, and unit-weighted composite scores were formed for planning, monitoring, and evaluation. Second, the measurement model was evaluated through CFA, reporting model-fit indices, standardized loadings, CR, and AVE. Third, differences among the three phases were tested using a Friedman test with follow-up paired-samples *t*-tests, and Pearson correlations quantified the associations among phases. Fourth, group differences were examined by gender (independent-samples *t*-tests, Cohen's *d*) and by frequency of ChatGPT use [one-way analysis of variance (ANOVA)]. Finally, to move beyond description, a multiple regression tested whether the three SRL phases predicted students' perceived writing outcomes. Statistical significance was set at *p* < 0.05.

#### Interview

Once the survey analysis was completed, an email was sent to participants to gauge students' interest in participating in the interview and to gain an in-depth understanding of ChatGPT's role in completing their dissertations. A total of 25 students expressed interest; only 20 were considered based on data saturation. A semi-structured protocol was used to gain in-depth information from the selected participants. Each interview lasted about 30–60 min per participant. The interviews were audio-recorded and transcribed verbatim. The interview questions are:

1. How do you plan your research or writing tasks when using AI tools for thesis writing? Please elaborate on your answers with examples.
2. How do you check or monitor the quality and accuracy of the AI-generated responses? Please detail your answers.
3. How do you evaluate whether AI use has improved your thesis writing?

Braun and Clarke's (2019) six-step thematic analysis guided the categorization of emerging themes for the interview. The steps involved familiarizing oneself with the data, generating initial codes, searching for themes, reviewing the themes, defining and naming the themes, and finally, producing reports. The authors chose manual coding to gain an in-depth understanding of phenomenology under investigation. First, the first author read the interview transcripts. Then they repeated and coded notes to develop familiarity with the dataset. Initial codes were generated by carefully reviewing the coding notes. This was followed by manual, line-by-line coding to generate initial codes. Codes with similar meanings were then collated and organized into categories and further refined into broader themes. Two trained coders independently analyzed the interview data, and any disagreements were resolved by employing a tiebreaker. A deductive approach was applied to categorize the themes according to planning and monitoring, employing intrinsic, extraneous, and germane loads.

#### Trustworthiness

This study followed the four criteria suggested by Guba and Lincoln (1994). For confirmability, member checking was conducted intermittently for each interview to ensure accurate interpretation of participants' responses. For credibility, member checking was conducted by asking participants to review the interview data for accuracy. Any inaccuracy in ideas is removed during member checking (Maxwell, 2005). Two independent coders were trained using a few examples from the data before the actual coding began. Inter-rater reliability was assessed using Miles and Huberman's (1994) raw percentage. The study scored an agreement of 90%. As for the qualitative data, the interview protocol was checked for weaknesses during the pilot study. Transferability was addressed by providing detailed descriptions of the participants, context, instruments, and research procedures (Erlandson, 1993).

## Findings

Planning is quite high, ranging from 3.63 to 4.29, indicating a strong agreement that helps them to plan their writing effectively. As highlighted by Table 2, the item “ChatGPT saves me time in my writing” has the highest rating with a score of 4.29, while the lowest mean score in this construct is

“I rely on ChatGPT to generate outlines or structures for my thesis writing.” (3.7)

**Table 2:** Descriptive statistics related to planning, monitoring and evaluation phases.

Planning	Mean	SD
1. I use ChatGPT to organize and plan my thesis.	4.168	0.733
2. ChatGPT helps me clarify my objectives for each stage of my study.	3.746	0.927
3. I rely on AI tools to generate outlines or structures for my thesis writing.	3.673	1.001
4. Using ChatGPT saves me time in planning my writing	4.290	0.692
5. I set specific goals before using ChatGPT for my academic work	4.188	0.686
Monitoring		
6. I use ChatGPT to check my progress during my research writing.	4.221	0.733
7. I compare my ideas with AI-generated suggestions to refine research writing.	4.112	1.010
8. ChatGPT helps me identify weaknesses or gaps in my writing	4.218	0.771
9. I regularly assess whether ChatGPT's feedback aligns with my research objectives.	3.818	0.985
10. I adjust my approach based on the feedback or insights I receive from AI tools.	4.195	0.699
Evaluating		
11. I evaluate the usefulness of AI's output before using it in my writing. 4.	4.185	0.709
12. I can critically distinguish between valid and invalid AI-generated information.	3.314	1.132
13. I reflect on how ChatGPT contributes to improving my research writing	4.099	0.859
14. I ensure that my final work maintains my own research	4.198	0.681
15. I review my AI-assisted work to check for originality and accuracy.	4.317	0.704

This indicates that students are not totally dependent on ChatGPT for their thesis writing.

For the monitoring construct, the item receiving the highest rating is “I use ChatGPT to check my progress during my research writing,” with a mean score of 4.2, indicating that ChatGPT provides valuable writing support. The lowest mean for this construct is “I regularly assess whether ChatGPT's feedback aligns with my research objective” with a mean score of 3.8. This is probably because students are not entirely dependent on ChatGPT and use it only when necessary. The construct related to the highest mean is the item “I review my AI-assisted work to check for originality and accuracy,” which indicates ethical and appropriate use of ChatGPT in their writing, with a mean score of 4.3. However, there is lower agreement for the item related to “I can critically distinguish between valid and invalid AI-generated information” with a mean score of 3.3. The lowest mean score can be interpreted as students' inability to critically distinguish the reliability of AI-generated ideas from their own.

### Composite dimension scores and comparison between SRL phases (RQ1)

To address RQ1 at the construct level, unit-weighted composites were computed for each phase. As shown in Table 3, all three phases were rated highly, with monitoring the strongest (*M* = 4.11, SD = 0.56), followed by evaluation (*M* = 4.02, SD = 0.56) and planning (*M* = 4.01, SD = 0.55). A Friedman test confirmed that ratings differed across phases, χ^2^ (2) = 38.94, *p* < 0.001. Follow-up paired-samples *t*-tests showed that monitoring was rated significantly higher than both planning, *t*(302) = 3.56, *p* < 0.001, d_z = 0.20, and evaluation, *t*(302) = 3.41, *p* < 0.001, d_z = 0.20, whereas planning and evaluation did not differ, *t*(302) = 0.33, *p* = 0.74. This pattern indicates that, while students engage all three phases, their regulatory activity concentrates on real-time monitoring of AI output.

**Table 3:** Composite descriptive statistics and reliability of the SRL sub-scales (*N* = 303).

SRL phase	Items	M	SD	α	CR	AVE
Planning	5	4.01	0.55	0.69	0.71	0.34
Monitoring	5	4.11	0.56	0.67	0.68	0.30
Evaluation	5	4.02	0.56	0.70	0.72	0.34
Overall SRL	15	4.05	0.48	0.86	–	–

The three phases were strongly and positively intercorrelated (planning–monitoring *r* = 0.61; planning–evaluation *r* = 0.61; monitoring–evaluation *r* = 0.66; all *p* < 0.001; Table 4), consistent with Zimmerman's (2000) conceptualization of SRL as an integrated, cyclical process rather than a set of independent skills.

**Table 4:** Pearson correlations among SRL phases.

	Planning	Monitoring	Evaluation
Planning	–		
Monitoring	0.61^**^	–	
Evaluation	0.61^**^	0.66^**^	–

^**^p < 0.001.

### Measurement model: confirmatory factor analysis

A three-factor CFA (planning, monitoring, evaluation) was estimated to verify whether the measurement model adequately represented the intended dimensions. The model showed acceptable but not very strong in Table 5. All standardized loadings were significant and ranged from 0.39 to 0.71. Composite reliability was adequate (CR = 0.68–0.72), but the average variance extracted fell below the 0.50 benchmark for each factor (AVE = 0.30–0.34), and the latent factors were very highly correlated (ϕ = 0.92–0.97). These results provide qualified support for the three-phase structure: the phases are empirically distinguishable yet share substantial variance, reinforcing the interpretation of SRL as a tightly coupled cyclical construct. The modest AVE and near-collinear factors are acknowledged as a measurement limitation, and a higher-order SRL model and item refinement are recommended for future validation.

**Table 5:** Confirmatory factor analysis fit indices for the three-factor SRL model.

Fit index	Value	Recommended
χ^2^ (df)	275.36 (87)	–
χ^2^/df	3.17	< 3–5
CFI	0.92	≥0.90
TLI	0.89	≥0.90
RMSEA	0.079	≤ 0.08
SRMR	0.081	≤ 0.08

CFI, comparative fit index; TLI, Tucker–Lewis index; RMSEA, root mean square error of approximation; SRMR, standardized root mean square residual.

### Group comparisons: gender and frequency of ChatGPT use

No statistically significant gender differences emerged on any phase or on overall SRL in Table 6 (all *p* ≥ 0.25; |*d*| ≤ 0.13), indicating that self-regulated use of ChatGPT in thesis writing was comparable for female and male postgraduates. In contrast, frequency of ChatGPT use was significantly associated with SRL. As shown in Table 7, a one-way ANOVA revealed reliable differences in overall SRL, *F*_(2, 300)_ = 24.63, *p* < 0.001, η^2^ = 0.14, and in every phase. Tukey's honestly significant difference (HSD) comparisons showed that daily (*M* = 4.12) and weekly (*M* = 4.01) users reported significantly higher SRL than occasional/rare users (*M* = 3.32; both *p* < 0.001), whereas daily and weekly users did not differ (*p* = 0.11). Thus more habitual engagement with ChatGPT was accompanied by stronger—not weaker—self-regulation, suggesting that regulatory skill and sustained use develop together.

**Table 6:** Gender differences in SRL (independent-samples *t*-tests).

SRL phase	Female M (SD)	Male M (SD)	*t*	*p*	*d*
Planning	4.01 (0.57)	4.02 (0.52)	−0.23	0.82	−0.03
Monitoring	4.12 (0.58)	4.10 (0.54)	0.34	0.73	0.04
Evaluation	4.06 (0.56)	3.98 (0.56)	1.16	0.25	0.13
Overall SRL	4.06 (0.50)	4.04 (0.46)	0.49	0.62	0.06

**Table 7:** Differences in SRL by frequency of ChatGPT use (one-way ANOVA).

SRL phase	Daily (*n* = 206)	Weekly (*n* = 81)	Occ./rare (*n* = 16)	*F*	*p*	η^2^
Planning	4.09	4.00	3.14	26.34	< 0.001	0.15
Monitoring	4.19	4.08	3.34	19.37	< 0.001	0.11
Evaluation	4.10	3.94	3.48	11.02	< 0.001	0.07
Overall SRL	4.12	4.01	3.32	24.63	< 0.001	0.14

### Explanatory analysis: SRL as a predictor of perceived writing outcomes

To move beyond description and test explanatory relationships, students' perceived writing outcomes (a six-item scale; α = 0.79; *M* = 4.11, SD = 0.54) were regressed on the three SRL phases. The model in Table 8 was strong, *R*^2^ = 0.63, *F*_(3, 299)_ = 171.9, *p* < 0.001: planning (β = 0.47), evaluation (β = 0.23), and monitoring (β = 0.18) each independently predicted perceived writing gains (all *p* < 0.001). At the composite level, SRL alone accounted for 61% of the variance in perceived outcomes (*r* = 0.78). This analysis directly addresses the concern that the study explains only “what” and not “why”: students who regulated their ChatGPT use more strongly, especially through deliberate planning—perceived greater improvement in their thesis writing, providing empirical support for the pedagogical value of scaffolding SRL rather than merely providing access to AI.

**Table 8:** Regression of perceived writing outcomes on SRL phases.

Predictor	Std. β	*p*	Model
Planning	0.47	< 0.001	*R*^2^ = 0.63
Monitoring	0.18	< 0.001	*F*_(3, 299)_ = 171.9
Evaluation	0.23	< 0.001	*p* < 0.001

### Mixed-methods integration: joint display

To integrate the two strands rather than report them in parallel, Table 9 presents a joint display that links each quantitative result to its corresponding qualitative theme and states the resulting meta-inference. The strands largely converge: high planning and monitoring scores are mirrored by accounts of AI-supported outlining and active verification. The most informative case is a complementarity relationship in the evaluation phase, where the low quantitative rating for distinguishing valid from invalid AI content (*M* = 3.31) is explained by the qualitative data—students compensate for limited confidence in judging AI reliability by manually tracing sources, cross-checking digital object identifiers (DOIs), and consulting supervisors. One point of divergence is also visible: although planning scored highly overall, a subset of interviewees described situational, troubleshooting use without deliberate goal setting, qualifying the quantitative picture of uniformly strategic planning.

**Table 9:** Joint display integrating quantitative and qualitative findings.

SRL phase	Quantitative result	Qualitative theme	Meta-inference
Planning	High agreement (*M* = 4.01); lowest item = reliance on AI outlines (*M* = 3.67)	AI-supported planning strategies; situational, troubleshooting use by some	Convergence: students plan with, but do not delegate to, AI—authorial control retained
Monitoring	Highest phase (*M* = 4.11); weakest item = checking alignment with objectives (*M* = 3.82)	Verification and triangulation; prompt refinement; cross-checking sources	Convergence: active metacognitive control dominates monitoring
Evaluation	Strong overall (*M* = 4.02) yet lowest single item = distinguishing valid vs. invalid AI content (*M* = 3.31)	Critical evaluation, ownership, ethical awareness; supervisor consultation to offset uncertainty	Complementarity: qualitative accounts of verification behavior explain the low validity-discrimination score—a targeted AI-literacy gap

#### Qualitative findings

The quantitative results indicated highly positive outcomes for the planning dimension, suggesting that participants engaged in goal setting, task analysis, and strategic planning with the support of ChatGPT. In Zimmerman's (2000) self-regulated learning framework, these processes correspond to the Forethought phase, where learners establish goals, analyse task requirements, and select strategies before engaging in the writing task. The following themes and sub-themes further triangulate the quantitative findings by illustrating how ChatGPT supported students' forethought processes during thesis preparation.

### Planning

The quantitative results indicated highly positive outcomes for the planning dimension, suggesting that participants were planning and organizing their learning tasks, as well as preparing their writing with the support of ChatGPT. These positive statistical findings indicate that ChatGPT played a significant role in facilitating learners' writing processes, particularly evident in enhancing their ability to plan and structure their academic work effectively. The following themes and sub-themes further triangulate the quantitative data, providing deeper insights.

The sub-themes that emerged for planning are outlining chapters, brainstorming ideas, and clarifying objectives at various stages. At the same time, there were sub-themes that highlighted students' irregular use, which was only for troubleshooting.

A number of participants reported that limited engagement with AI during their initial stages was only a guiding tool, not a central tool controlling their ideas. One of the participants said, “I use AI tools mainly to outline each chapter and identify key points I need to address” (T2). Another participant added that it is meant for brainstorming. For T2, it is “only used for brainstorming and not the whole thesis,” demonstrating that AI was primarily used to generate ideas rather than to write the entire thesis. T6 said that “ChatGPT transformed scattered entries into a coherent, logically connected argument, cutting revision time in half”, indicating ChatGPT's ability to save time.

Similarly, T12 said:

I usually make an outline by myself first. “I don't ask AI to write a thesis for me. I plan. For example, Today I want to write the section about concrete durability. Then I ask AI to give me a structure for this specific section. It helps me fill in the blanks.” (T15)

The participants' findings indicate that ChatGPT was primarily used as a support tool to clarify specific objectives in their chapters. At the initial stage, one participant said that ChatGPT was used for conceptual orientation, stating, “like interpreting analysis of covariance (ANCOVA) results. I asked ChatGPT to check my report” (T22), which reveals that the tool assists in identifying relevant sources and refining the study's focus for each chapter. As the research progressed, ChatGPT continued to support students by clarifying their methodology chapter. One participant noted, “When I began to write Chapter 3, I gave the information to AI. It told me that the proper research design is a quasi-experimental non-equivalent control group design” (T12), demonstrating that ChatGPT assisted in aligning methodological decisions with research aims. For the literature review, T17 said:

I mainly use AI tools when I need to search for literature. For example, I will ask the AI to help me identify key papers, summarize the main arguments of several articles, or suggest related keywords so that I can search more effectively in academic databases.

At later stages, ChatGPT was used to verify and refine analytical and interpretive objectives. A participant mentioned using it “when writing chapters 4 and 5, I had to make sure the responses tally with what the paper requires” (T18), reflecting its role in validating findings and discussion. Similarly, another participant highlighted its usefulness during analysis, stating:

I will ask the AI to analyse and refine the data. For instance, when drafting sections related to data analysis, I might instruct ChatGPT: “Validate the structural equations of the variables. If any reliability or validity metrics fall short, please advise on how to revise the questionnaire content.” This allows me to anticipate potential issues through AI simulation and adjust accordingly.

While many participants highlighted the effective use of ChatGPT, some participants did not use it regularly. T4 explained, “Actually, I do not have a clear plan when using AI tools. They are often employed when I encounter some difficulties.” This excerpt demonstrates that there is no plan to use AI; it is only used as a troubleshooting tool, not as a planned scaffolding tool. T12 expressed that “I don't think AI helps me clarify my research ideas, since I need to give AI a command, and then give it further information until the ideas are clear.” This excerpt reflects a trial-and-error interaction process, where feedback are not blatantly given and goes through a number of promptings. This process points to the fact that cognitive effort is without strategic pre-planning.

Similarly, another participant voiced out:

When I order AI to write a framework or proposal for me, I don't think it can deliver a very comprehensive one. I need to make further corrections; it can still be a good proposal. While this must be based on every detail of proposal writing and your rich knowledge. Otherwise, you don't even know how to interact with AI, and you cannot adjust your approach.

Collectively, the excerpts highlighted that ChatGPT can serve as a cognitive scaffolding tool, helping students clarify and align their objectives across multiple stages of the research process. At the same time, the findings also suggest that AI use, for some of the participants, is situational and problem-solving rather than integrated in their writing for the self-regulated learning cycle. The lack of planning by postgraduates in their dissertation writing suggests that ChatGPT has not been used regularly. Although students still exercised control by adjusting prompts and providing additional information, the interaction appears emergent rather than strategically structured. This theme highlights variability in self-regulated AI use, demonstrating that not all postgraduate students engage with AI through deliberate planning or proactive goal setting.

Viewed through Zimmerman's SRL framework, these findings indicate that ChatGPT primarily supported the Forethought phase by helping students formulate goals, organize thesis chapters, identify relevant literature, and clarify task requirements before writing. However, the findings also reveal differences in students' strategic planning, with some participants using ChatGPT systematically as a planning tool while others engaged with it only when difficulties emerged. This distinction highlights varying levels of forethought regulation among postgraduate students.

#### Monitoring in strategy adjustment

In Zimmerman's SRL model, the Performance phase involves self-monitoring, strategy implementation, and ongoing adjustments while completing a task. The quantitative findings indicate satisfaction with ChatGPT's role during writing, and the qualitative findings further demonstrate how participants monitored their progress, evaluated AI-generated responses, and refined their strategies through iterative interactions with ChatGPT.

The quantitative findings indicate satisfaction, and the following sub-themes triangulate them. The emerging subtheme is verifying information for triangulation. The data reveal that participants underwent a monitoring phase during which AI was used to support their writing. The participants seem critical when using AI tools and tend to use them to verify and triangulate information. For example,

When drafting my literature review, AI initially produced content that was too general, so I adjusted my approach by providing more specific keywords and clearer instructions. This helped generate more relevant and academically appropriate material for my thesis.

AI is used for self-checking ideas and information needed for a dissertation.

Similarly, another T7 said that:

When I ask AI tools to paraphrase a paragraph first, and the results are unsatisfactory, I will adjust my prompts to ask it to refine again, ensuring readability and persuasiveness.

T19 suggested that the tool was employed to compare and cross-check information from multiple sources, thereby strengthening confidence in their academic arguments. T12 explained:

When using AI tools for literature-related tasks, I always verify the information manually. For example, if the AI provides a DOI, I will search for it myself to confirm that the article exists and that the bibliographic details are correct. I also double-check the accuracy of the citations to ensure the AI's summary or interpretation matches the original source.

For T12:

I evaluate improvement by comparing earlier drafts with AI-assisted versions to see whether the clarity, structure, and grammar have become more polished. I also check whether the writing flows more logically and whether sections that were previously unclear have become easier to understand.

One student remarked, “ChatGPT might misunderstand my intention, so I had to adjust my words to make it understand” (T20). T10 said, “I will read one by one to make it consistent with my previous content, rather than directly copying it”.

This excerpt indicates a metacognitive process in which learners evaluate AI, evaluate the AI's responses, and generate ideas and information. Overall, these findings suggest that postgraduate students monitor their writing while considering AI by verifying the content, triangulating information, and refining their writing. In other words, metacognitive control is taking place when integrating AI into their writing.

These findings align closely with Zimmerman's Performance phase because participants actively monitored the quality of AI-generated content, adjusted prompts when responses were unsatisfactory, cross-checked information against external sources, and revised their writing accordingly. Rather than accepting AI outputs passively, students demonstrated ongoing self-regulation through continuous monitoring and strategic adaptation during the writing process.

#### Self-reflection phase: evaluation and self-judgement

Zimmerman conceptualizes the Self-Reflection phase as the stage in which learners evaluate their performance, judge the effectiveness of their strategies, and reflect on outcomes. The sub-themes related to evaluation are critical review before adoption, maintaining ownership, and strategic integration. These themes reflect participants' efforts to assess the value of AI-generated content while preserving their academic identity and authorship.

The sub-themes related to evaluation are critical review before adoption, maintaining ownership, and strategic integration. The above theme reflects the participant's ability to carefully scrutinize the credibility of AI content before incorporating it in their writing. For example, “I critically review my own writing.” Participants tend to reflect on AI output and ask their supervisors for guidance. T6 explained that:

To evaluate whether AI has improved my thesis writing, I often ask my supervisor to review my work. Their feedback helps me understand whether the writing is clear and well-structured, and whether each subsection effectively conveys its main points. By comparing my drafts before and after using AI assistance, I can also see whether the writing becomes more organized and aligned with academic expectations.

Participants do not consider AI output; they focus on how it supports their writing. In T7′words:

I evaluate improvement by comparing earlier drafts with AI-assisted versions to see whether the clarity, structure, and grammar have become more polished. I also check whether the writing flows more logically and whether sections that were previously unclear have become easier to understand.

T12 explains:

I always start by forming my own arguments and outlines before using any AI tools, so the core ideas come from me. I also critically revise AI-generated suggestions, keeping only the parts that align with my reasoning and rewriting sections to ensure they reflect my analytical perspective.

The findings demonstrate that participants control their writing. AI is merely an assistive tool, and AI does not complete theses. Participants have the opportunity to practice metacognitive control and engage in academic negotiations with AI tools. T19 asserts that “My experience and common sense, sometimes I will trace the references to see if they are current or correct”.

Another participant claims that “I only generate the draft using AI, other than that I write and elaborate my own points for my work to ensure it's written in my own voice” (T1), highlighting the fact that his/her ideas were produced and followed by interaction with AI to verify the information. This demonstrates an agentic and self-regulated approach, in which participants decide both their writing and its content.

Importantly, these reflective judgments extend beyond simple verification of AI outputs. Participants evaluated whether ChatGPT genuinely improved their writing quality, considered the extent to which AI-supported revisions aligned with their academic intentions, and reflected on the appropriateness of incorporating AI-generated suggestions into their thesis work. Such judgments represent core characteristics of Zimmerman's Self-Reflection phase.

Participants acknowledge the risk of being overly dependent on AI. According to T9:

I am always careful with AI-generated answers. I verify the information by checking original sources one by one. I also compare across multiple credible references to confirm consistency. If something looks doubtful, I flag it and rewrite or remove it.

To sum up, the findings suggest that participants actively safeguarded their authorship by critically evaluating AI-generated content and reflecting on its contribution to their writing. Consistent with Zimmerman's Self-Reflection phase, participants assessed the effectiveness of AI-assisted strategies, maintained ownership of their academic work, and made informed decisions regarding the integration of AI into their thesis writing. This ensured that their dissertations remained grounded in their own scholarly contributions while benefiting from AI-supported assistance.

The summary of the themes and sub-themes is provided in Table 10.

**Table 10:** Summary of themes and sub-themes.

SRL phase	Theme	Sub-theme	Representative evidence
Planning	Forethought Phase (Goal setting, Task Analysis, strategic Planning)	Outlining chapters	“I use AI tools mainly to outline each chapter and identify key points I need to address.” (T2)
		Brainstorming ideas	“It is only used for brainstorming and not the whole thesis.” (T2)
		Clarifying research objectives	“I ask AI to give me a structure for this specific section. It helps me fill in the blanks.” (T15)
		Literature search and conceptual clarification	“I ask the AI to identify key papers or suggest keywords so that I can search more effectively.” (T17)
		Troubleshooting during writing difficulties	“I do not have a clear plan when using AI tools. They are often employed when I encounter difficulties.” (T4)
Monitoring	Performance Phase (Self- Monitoring and Strategy Adjustment)	Checking accuracy of AI output	“If the AI provides a DOI, I search for it myself to confirm the article exists.” (T12)
		Prompt refinement	“If the results are unsatisfactory, I adjust my prompts to refine the paragraph.” (T7)
		Comparing multiple sources	“I compare information from multiple sources to strengthen my arguments.” (T19)
Evaluating	Self-Reflective Phase (Self Evaluation and Reflective Judgement)	Reviewing AI-assisted drafts	“I compare earlier drafts with AI-assisted versions to see if the clarity improves.” (T7)
		Preserving authorial voice	“I start with my own arguments and revise AI suggestions to reflect my perspective.” (T12)
		AI as supportive rather than replacement tool	“I only generate the draft using AI, but I elaborate my own points to ensure my own voice.” (T1)
		Verifying references and avoiding over-reliance	“I verify the information by checking original sources one by one.” (T9)

## Discussion

The above excerpts suggest that the use of AI is a personal matter for refining and confirming structural decisions. The current study examined how SRL can assist students in using ChatGPT pedagogically to guide them in writing their chapters, along with their writing experiences and the factors that influence or hinder them. The quantitative findings demonstrate that postgraduates were actively involved in the three important phases of self-regulated learning, particularly planning, monitoring, and evaluation, when using AI in their thesis writing. ChatGPT was used as an assistive tool wisely, with an effective strategy and evaluation behavior as suggested by Zimmerman's (2000) framework. This aligns with previous studies that demonstrate that ChatGPT enhances self-regulation among learners (Chiu, 2024a; Ng et al., 2024; Zhu, 2025). Therefore, it can be inferred that AI is a user-friendly assistive tool for applying self-regulated strategies during the planning, monitoring, and evaluation phases. Figure 1 illustrates the integration of AI tools within Zimmerman's (2000) cyclical model of SRL, consisting of three interrelated phases: planning, monitoring, and evaluation. The model demonstrates the iterative nature of SRL in the use of AI in writing a thesis.

**Figure 1:**
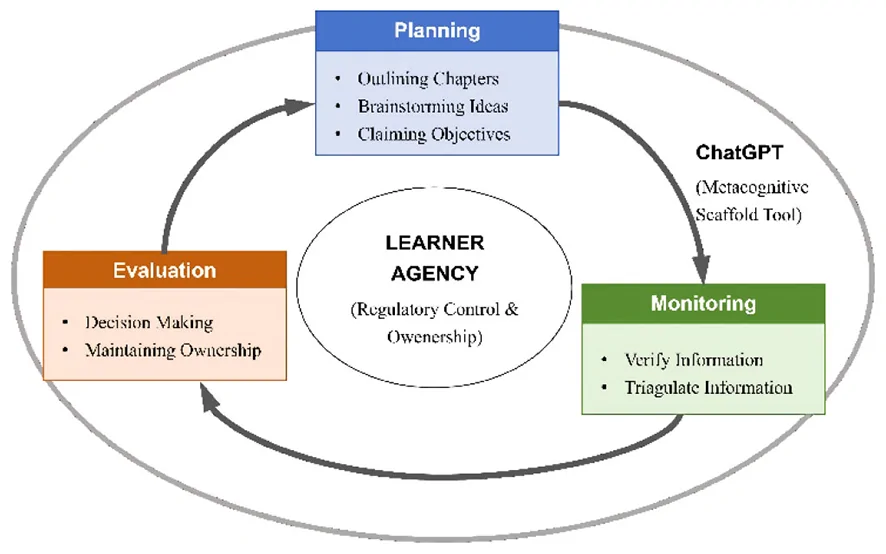
ChatGPT as a metacognitive scaffold supporting learner agency in self-regulated thesis writing.

One notable finding from the quantitative results is that students reported the lowest level of agreement for the item measuring their ability to critically distinguish between valid and invalid AI-generated information (*M* = 3.31). This finding is particularly significant because it reveals a potential gap in AI literacy among postgraduate students. While the results indicate that students demonstrate relatively strong self-regulated behaviors in terms of planning and monitoring their writing processes, their ability to critically evaluate the credibility of AI-generated information appears to be comparatively weaker.

This finding highlights an important challenge in AI-assisted learning environments. Although generative AI tools such as ChatGPT can effectively support idea generation, writing organization, and feedback, learners may still struggle to assess the reliability and validity of AI-generated content. Such limitations have also been noted in previous research, which warns that AI-generated outputs may contain inaccuracies or fabricated references. Therefore, students must develop stronger critical evaluation skills when interacting with AI systems.

From a self-regulated learning perspective, this finding suggests that the evaluation phase of SRL may require an additional layer of cognitive regulation in AI-mediated learning contexts. In addition to reflecting on the quality of their own writing, learners must now engage in source verification and credibility assessment of AI-generated information. This finding extends Zimmerman's (2000) SRL framework by suggesting that learners are no longer regulating only their own learning behaviors and academic products but are also required to regulate externally generated knowledge produced by AI systems. In traditional SRL contexts, evaluation focuses primarily on self-performance and task outcomes; however, in AI-supported environments, learners must additionally assess the trustworthiness, accuracy, and academic appropriateness of machine-generated content. This represents a form of dual regulation in which students simultaneously regulate their learning processes and the outputs of AI tools. Consequently, AI literacy becomes an integral component of effective SRL in contemporary academic writing contexts.

The qualitative data revealed that the planning phase allowed students to brainstorm and outline the structure of specific chapters of their thesis. Findings revealed that they employed ChatGPT for clarifying the research objectives of certain of their thesis chapters, literature search and methodological decisions. Also, some participants noted that they set certain goals before using AI tools. It is used as a scaffolding tool, not as an intellectual tool to write their dissertation. This finding is consistent with Kuhail et al. (2023)'s idea that optimal use of AI is very likely to depend on applying AI output critically and effective questioning skills. Beyond confirming previous research, the findings provide theoretical support for Zimmerman's forethought phase by demonstrating how AI can function as a metacognitive scaffold that facilitates goal setting, strategic planning, and task analysis. Rather than replacing learners' planning processes, ChatGPT appeared to amplify them by enabling students to explore alternative directions, clarify research intentions, and refine writing goals before engaging in the actual writing task. This suggests that AI can strengthen learner agency when used strategically, supporting the argument that SRL remains learner-driven even in technology-rich environments.

The monitoring phase revealed that participants were actively verifying ideas and information, triangulating ideas, refining their writing, and adjusting prompts when ChatGPT's outputs were inaccurate. This implies learner metacognitive behavior, where learners compare and assess the credibility of information produced by AI. Instead of passively accepting AI, participants are actively making critical judgments in their writing. Liu et al. (2021) similarly discovered that AI use in the educational context promotes greater self-monitoring. This finding confirms a central assumption of SRL theory that effective learners continuously monitor their progress and adapt their strategies when necessary. Importantly, the findings challenge common concerns that AI inevitably encourages passive learning. Instead, participants demonstrated active engagement through iterative prompting, verification of information, and refinement of outputs. These behaviors reflect metacognitive monitoring and strategic adaptation, suggesting that AI-supported writing can promote higher-order regulation when learners critically engage with the technology rather than rely on it uncritically.

The evaluation phase demonstrates participants' positive behavior of reviewing AI by using their personal knowledge to decide on the AI output. The decisions were made entirely by the participant, with attention to clarity, accuracy, and the participant's own academic voice. More importantly, participants have also consulted with their instructors for further clarification. This ensures their dissertation writing represents their own scholarly contribution, with a very strong sense of ethics and integrity throughout their journey in completing it. Limitations of AI and cultivating critical thinking during use to maintain ownership of their writing and to be independent, as suggested by Sullivan et al. (2023). Werdiningsih et al. (2024) assert that frequent AI support made some students feel detached from their writing identity, perceiving AI-generated phrasing as overly formulaic. The findings further extend the self-reflection phase of SRL by demonstrating that evaluation in AI-supported writing is not limited to judging the quality of one's own work. Students also evaluated whether AI-generated suggestions aligned with disciplinary expectations, supervisory feedback, and their own academic identity. This finding introduces an important theoretical dimension to SRL in AI-mediated learning, namely identity regulation. Participants actively filtered AI-generated content to preserve their scholarly voice and intellectual ownership of their theses. Such behavior suggests that successful AI-supported learning involves not only cognitive and metacognitive regulation but also the regulation of authorship and academic integrity.

While acknowledging SRL, participants seem aware of the challenges of using ChatGPT for thesis writing. Such an attitude serves as an intelligent mechanism against over-dependence on AI and imbalance, and for the wise use of AI integration in their writing process. In a way, students tend to exercise control over their writing, which defines an important characteristic of SRL. The findings demonstrate the agentic use of AI in dissertation writing, an important skill that postgraduates need to cultivate. It is pertinent for students to have adequate knowledge of AI's strengths and weaknesses to think critically and wisely, thereby enhancing learning (Ng et al., 2024; Xia et al., 2024). These findings also suggest that generative AI tools such as ChatGPT may function as adaptive elements within smart learning environments, supporting learners' metacognitive regulation and reflective engagement during complex academic writing tasks. Theoretically, these findings support and extend existing SRL literature by demonstrating that learner agency remains central even when advanced AI tools are incorporated into academic writing. Rather than diminishing self-regulation, ChatGPT appears to create new opportunities for learners to exercise strategic control, metacognitive awareness, and reflective judgment. This finding challenges deterministic perspectives that portray AI as replacing human cognition and instead positions AI as a metacognitive partner whose educational value depends on the learner's capacity for self-regulation. Consequently, the study contributes to emerging scholarship on AI-supported learning by proposing that SRL functions as a key mechanism through which students transform AI assistance into meaningful academic outcomes.

In sum, the current study significantly contributes to the literature related to SRL and AI in higher education institutions. It can be deduced that SRL is pertinent in AI-assisted academic environments. All in all, the findings suggest that AI can be employed wisely by postgraduate students based on SRL; when used systematically, it can encourage metacognitive engagement. Rather than denying the use of AI, it is important to embed AI within an effective framework of planning, monitoring, and evaluation. By fostering learners' agency and critical evaluation, AI can assist students in developing their theses effectively. This positive attitude is in line with Sabzalieva and Valentini (2023) emphasis that “the algorithm by which it works does not take a definite position, as its interpretation is based on the statistical analysis of billions of texts on the Internet” (p. 5). Since AI-generated information is based on probabilistic patterns rather than accurate judgment. Consequently, students carefully evaluate AI-generated content and verify its accuracy so that their dissertations reflect their own academic voice.

### Group-level and predictive patterns

Three inferential findings extend the descriptive account. First, the absence of gender differences suggests that self-regulated AI use in thesis writing is not gendered at this postgraduate level, and that pedagogical support need not be differentiated by gender. Second, the positive association between frequency of ChatGPT use and SRL indicates that more habitual users regulated their engagement more, not less, tempering fears that frequent AI use erodes learner agency; the direction of this relationship, however, cannot be established from cross-sectional data and warrants longitudinal testing. Third, the regression showing that the three SRL phases jointly explained 63% of the variance in perceived writing gains—with planning the strongest predictor—provides explanatory evidence that regulated, deliberate AI use, rather than access alone, is what students associate with improvement. Together these results answer the reviewers' call to move from “what was found” toward “why,” positioning SRL as a mechanism that links AI use to writing outcomes.

## Pedagogical implication

The findings of this study offer important pedagogical implications for integrating ChatGPT into higher education, particularly in research writing contexts. It is pertinent to position AI scaffold postgraduate writing. Explicit instruction from instructors on the planning, monitoring, and evaluation phases will enable postgraduates to use AI effectively. This will allow postgraduates to intentionally employ AI tools rather than engage with them. Educators stress the importance of self-checking and triangulation is pertinent to reinforce critical literacy. Educators should emphasize the importance of postgraduate students deciding on their authorship and ownership of their research writing. Students need to be deeply engaged in critical thinking to adapt, integrate, and wisely reject ideas and information generated by AI tools.

## Theoretical contribution

This study contributes to the body of research on AI and self-regulated learning (SRL) by empirically situating AI use within the three significant phases of SRL: planning, monitoring, and evaluation. While previous studies have documented. AI as a cognitive support tool, the current study examines how learners actively engage with AI (ChatGPT) to enhance their thesis writing. Instructors can guide postgraduates with structured guidance to use AI to complete their dissertations.

Furthermore, the study extends SRL theory by showing that in AI-mediated learning contexts, the evaluation phase involves an additional layer of source verification and critical assessment of AI-generated information. This indicates that effective self-regulated learning in AI-supported academic writing requires not only regulating one's own learning processes but also critically regulating interactions with AI systems. This study highlights how learners actively regulate their writing processes when interacting with AI. The findings suggest that ChatGPT can function as a metacognitive scaffolding tool that supports learners in the planning, monitoring, and evaluation phases of SRL.

At the planning phase, AI is viewed as a scaffolding tool for organizing the dissertation chapters and identifying the objectives of each chapter. This extends the SRL framework by demonstrating how ChatGPT can support the processes, particularly in planning and structuring their chapters. Nevertheless, the findings also suggest selective use, reminding users that ChatGPT is not without limitations. They operate as a metacognitive aid. This finding refines the framework by placing AI (ChatGPT) as a conditional support rather than a deterministic support mechanism and not a determining force in their writing process.

During the monitoring phase, students used ChatGPT for self-checking and cross-checking information, demonstrating active metacognitive control rather than passively accepting ideas generated by ChatGPT. Such findings strengthen the monitoring phase by positioning AI as a tool for verifying and validating information against other sources.

At the evaluation phase, students demonstrated critical decision-making before adopting AI-generated suggestions and emphasized maintaining ownership of their academic voice. This finding contributes to SRL theory by highlighting the role of agency and authorship in AI-mediated learning, suggesting that evaluation in digital contexts involves not only accuracy checking but also identity regulation.

In conclusion, the learners remain at the center of the cycle, emphasizing that they are in control of their writing. ChatGPT is a metacognitive scaffolding tool and does not determine learning outcomes.

## Limitations and conclusion

The study explored the role of ChatGPT in guiding postgraduate students in writing their theses, focusing on SRL and its implications for pedagogy. The study discovered that ChatGPT assists students in SRL. Nevertheless, students are cautious when using ChatGPT, as they are aware of its tendency to hallucinate and to avoid overreliance. This implies that students tend to be critical of AI use. At this point, ChatGPT demonstrates its worth, offering a range of guides on aspects related to planning, monitoring, and evaluation phases. The outcome of this study contributes to understanding how ChatGPT and other AI tools can support or complicate thesis writing. The findings of this study not only support thesis writing but also serve as a substitute for traditional thesis writing. It is rather an assistive tool and conditional support for post-graduate students.

This study has several limitations. First, the use of purposive sampling in a single higher education institution in Malaysia limits the generalizability of the findings to other institutions and nations. The findings may also not apply directly to undergraduate students, whose academic writing tasks and levels of research independence may be different from those of postgraduate students. Second, as the study relied on self-reported survey and interview data, some participants may have overstated desirable behaviors such as source verification and critical evaluation. Third, the cross-sectional design does not allow causal conclusions regarding the relationships among ChatGPT use and self-regulated learning. Finally, considering ChatGPT and other generative AI tools develop rapidly, the findings may vary across model versions of AI, AI platform features, and institutional policies. This study can be more extensively applied in future studies to examine the impact of demographic, gender, age, moderators, use of technology and habitual use. Besides future research could include longitudinal designs in different sites and platforms to investigate behavior patterns or writing performances.

## Data Availability

The original contributions presented in the study are included in the article/supplementary material, further inquiries can be directed to the corresponding author/s.
